# Feasibility of ^31^P spectroscopic imaging at 7 T in lung carcinoma patients

**DOI:** 10.1002/nbm.4204

**Published:** 2019-11-17

**Authors:** Quincy (.Q.). van Houtum, Firdaus (.F.A.A.). Mohamed Hoesein, Joost (.J.J.C.). Verhoeff, Peter (.P.S.N.). van Rossum, Anne (.A.S.R.). van Lindert, Tijl (.T.A.). van der Velden, Wybe (.W.J.M.). van der Kemp, Dennis (.D.W.J.). Klomp, Catalina (.C.S.). Arteaga de Castro

**Affiliations:** ^1^ Radiology Department University Medical Center Utrecht Netherlands; ^2^ Radiotherapy Department University Medical Center Utrecht Netherlands; ^3^ Respiratory Medicine Department University Medical Center Utrecht Netherlands

**Keywords:** ^31^P MR spectroscopic imaging, *In vivo* application, lung carcinoma, response monitoring, X‐nuclei MRS

## Abstract

Currently, it is difficult to predict effective therapy response to molecular therapies for the treatment of lung cancer based solely on anatomical images. ^31^P MR spectroscopic imaging could provide as a non‐invasive method to monitor potential biomarkers for early therapy evaluation, a necessity to improve personalized care and reduce cost. However, surface coils limit the imaging volume in conventional ^31^P MRSI. High‐energetic adiabatic RF pulses are required to achieve flip angle homogeneity but lead to high SAR. Birdcage coils permit use of conventional amplitude modulated pulses, even over large FOV, potentially decreasing overall SAR massively. Here, we investigate the feasibility of 3D ^31^P MRSI at 7 T in lung carcinoma patients using an integrated ^31^P birdcage body coil in combination with either a dual‐coil or a 16‐channel receiver.

Simulations showed a maximum decrease in SNR per unit of time of 8% for flip angle deviations in short TR low flip‐angle excitation 3D CSI. The minimal SNR loss allowed for fast 3D CSI without time‐consuming calibration steps (>10:00 min.). ^31^P spectra from four lung carcinoma patients were acquired within 29:00 minutes and with high SNR using both receivers. The latter allowed discrimination of individual phosphodiesters, inorganic phosphate, phosphocreatine and ATP. The receiver array allowed for an increased FOV compared to the dual‐coil receiver.

3D ^31^P‐CSI were acquired successfully in four lung carcinoma patients using the integrated ^31^P body coil at ultra‐high field. The increased spectral resolution at 7 T allowed differentiation of multiple ^31^P metabolites related to phospholipid and energy metabolism. Simulations provide motivation to exclude ^31^P B_1_ calibrations, potentially decreasing total scan duration. Employing large receiver arrays improves the field of view allowing for full organ coverage. ^31^P MRSI is feasible in lung carcinoma patients and has potential as a non‐invasive method for monitoring personalized therapy response in lung tumors**.**

## Graphical abstract

The feasibility of ^31^P MRSI was demonstrated in four lung carcinoma patients using a ^31^P body coil in combination with a two‐ or 16‐channel receiver at 7 T. A maximum decrease of 8% SNR/unit of time from simulations motivated excluding B_1_ calibrations in fast 3D CSI. With improved MRI, ^31^P MRSI potentially provides a non‐invasive method to monitor biomarkers for early therapy evaluation, aiming for improved personalized care and reduced cost in lung tumor therapy.

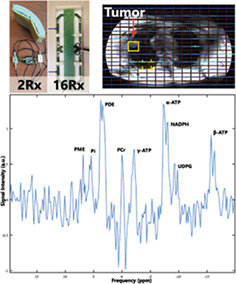

**Abbreviations**
^31^P: Phosphorus
ATP: Adenosine triphosphate
CSI: Chemical Shift Imaging
FID: Free induction decay
GPC: Glycerophosphocholine
GPE: Glycerophosphoethanolamine
MRSI: Magnetic Resonance Spectroscopic Imaging
NADPH: Nicotinamide adenine dinucleotide phosphate
PC: Phosphocholine
PCr: Phosphocreatine
PDE: Phosphodiesters
PE: Phosphoethanolamine
Pi: Inorganic Phosphate
PME: Phosphomonoesters
Rx: Receiver
SAR: specific absorption rate
TR: repetition time
UDPG: uridine diphosphoglucose

## 1 INTRODUCTION

In recent years, many new molecular therapies, such as immunotherapy, have been introduced for the treatment of lung cancer ^1^. Tumor cells generally use antigens to mask themselves from the immune system and immunotherapy exploits this mechanism by administering antibodies which specifically target tumor antigens. This labels the cell which allows it to be recognized by the own defense mechanisms of the body. The immune system responds by inhibiting or attacking the tumor cells, resulting in stalled tumor growth not necessarily accompanied by a decrease of tumor volume on imaging modalities ^2^.

Currently, it is difficult to predict which patients show an effective response to immunotherapy based on anatomical images like computed tomography only. Although a promising new treatment strategy for non‐small cell lung carcinoma, immunotherapy is expensive and severe drug side effects are observed accompanied by an apparent decrease in quality of life. Therefore, there is an unmet need for a non‐invasive method that can be used to predict tumor metabolic response which is crucial for early therapy effect evaluation. By adjusting the therapy strategy accordingly, such a tool would allow for more personalized curative care with less side effects, and reduced costs.

A recent study in breast cancer showed that changes in the phospholipid metabolism in responsive tumors can be detected after a single chemotherapy session using 31‐phosphorous (^31^P) magnetic resonance spectroscopic imaging (MRSI) ^3^, ^4^. ^31^P MRSI can detect the phospholipid and energy metabolites, which provides possibilities to monitor tissue metabolism non‐invasively during treatment. Inorganic phosphate (Pi), phosphocreatine (PCr) and ATP (with the α‐, β‐ and γ‐ resonances) allow assessment of the energy metabolism and the phosphomonoesters (PME) and phosphodiesters (PDE) provide insight into the phospholipid metabolism ^4^, ^5^, ^6^, ^7^. Enhanced ratios of phosphocholine (PC) to glycerophosphocholine (GPC) and phosphoethanolamine (PE) to glycerophosphoethanolamine (GPE), are frequently observed in tumor tissue and correlated with proliferation ^7^, ^8^, ^9^, ^10^, ^11^, ^12^. Another study in breast cancer demonstrated the feasibility of the phospholipid metabolism as biomarker for therapy follow‐up and additionally reported shortening of the transverse relaxation time of Pi as a biomarker ^4^, ^8^, ^13^. As the physiological changes are present before any morphological changes have occurred, these metabolites, their ratios and individual MR properties are potential (bio‐)markers for therapy response monitoring ^14^, ^15^, ^16^.

However, the individual detection of 31P metabolites is hampered at lower magnetic field strengths (3 T and below) due to the restricted spectral bandwidth and the low detection sensitivity. By going to higher magnetic field strengths (e.g. 7 T and higher), the SNR and spectral resolution are intrinsically enhanced ^17^, ^18^. These properties have a tremendous advantage for the low abundant ^31^P metabolites and even allow detection of the individual phosphomonoesters, (i.e. PE, PC) and diesters (i.e. GPE, GPC) ^19^.

Unfortunately, the imaging volume in conventional ^31^P MRSI is limited as small birdcage or surface coils are used ^5^, ^20^. Surface coils generally require the use of high‐energetic adiabatic RF pulses to achieve flip angle homogeneity as inhomogeneous excitations lead to signal variation in the acquired spectra over the large field of view. Adiabatic RF pulses usually result in high specific absorption rates (SAR), leading to longer repetition times (TR), clinically impractical scan times for a single protocol and a limiting number of consecutive scans. Full spectroscopic coverage of large organs such as the lungs is therefore challenging due to inhomogeneous B_1_^+/−^ fields and inhomogeneous excitation which increase with magnetic field strength.

In addition, MR imaging and spectroscopy are challenging near the lungs due to the presence of air, the relatively small amount of tissue and respiratory motion. Yet, previous studies claim that from a technical point of view MR imaging on clinical field strengths is a feasible method for screening lung cancer ^21^.

Recent studies from Loring et al. and van Houtum et al. have presented a ^31^P whole‐body birdcage coil designed for 7 T ^22^, ^23^. Using the body coil in combination with the conventional adiabatic pulses for high and low flip angle excitations requires adiabatic half passage or BIR4 pulses respectively and would increase the cost effective B_1_^+^. This results in a narrow band width leading to multiple acquisition to capture the full spectra. By design this coil results in an improved homogeneous excitation, comparable to the ^1^H whole‐body birdcage coils of clinical 3 T MR systems. This allows the use of rectangular pulses, which decreases global and local SAR, creating opportunities for fast spectroscopic imaging methods. In addition, they demonstrated that this ^31^P‐body coil even allowed quantification of transverse relaxation times and the feasibility of obtaining high flip angle chemical shift imaging (CSI), over a large field of view. However, the use of this coil was revealed with a 30% inter‐subject variation of the flip angle using a single power setting for multiple volunteers. This raises questions for the need for individual ^31^P calibration, especially at low flip angles, as only the effective flip angle and not B_1_^+^‐field homogeneity is affected. Low flip angle excitations accompanied with short repetition times (TR) can be used for fast 3D CSI. The optimal SNR per unit of time at lower flip angles is acquired when the Ernst angle (α_E_) is used and any deviation from this flip angle result in additional T_1_ weighting and a lower SNR per unit of time ^24^. The effects of a 30% flip angle deviation to the SNR per unit of time and consequently the acquired spectra can be evaluated by simulations. Excluding B_1_ calibrations can decrease the total scan duration by 10 minutes or more, subsequently increasing patient comfort or allowing for additional scans or additional sampled averages to improve SNR. ^25^

The primary aim of this study was to investigate the feasibility of 3D ^31^P MR spectroscopic imaging at ultra‐high field in combination with a ^31^P whole‐body birdcage coil in four lung carcinomas.

## 2 MATERIALS & METHODS

### 2.1 Simulations

The effect of an uncalibrated excitation, that leads to a deviation from the Ernst angle (α_E_) was assessed by simulating the SNR per unit of time for the α_E_ and for α_Ε_ with a ± 30% and ± 50% deviation over a TR/T_1_‐ratios range of 10^−6^ to 0.3. The latter is chosen with respect to a short TR of 60 ms and the longitudinal relaxation times (T_1_) for ^31^P metabolites of interest possibly ranging from 450 ms (α‐ATP) to 7000 ms (GPE) ^6^. The simulated spectroscopy signal shown in equation [1] was corrected for time differences by dividing with the square root of TR. The SNR per unit of time for all the calculated TR/T1‐ratios were normalized to the maximum signal at α_E_.

(1) $$\text{signal}\propto \frac{\sin\left(\alpha \right)\;\left(1-e^{-\frac{\mathrm{TR}}{T_{1}}}\right)}{1-\cos\left(\alpha \right)\;e^{-\frac{\mathrm{TR}}{T_{1}}}}$$

### 2.2 Materials

**FIGURE 1:**
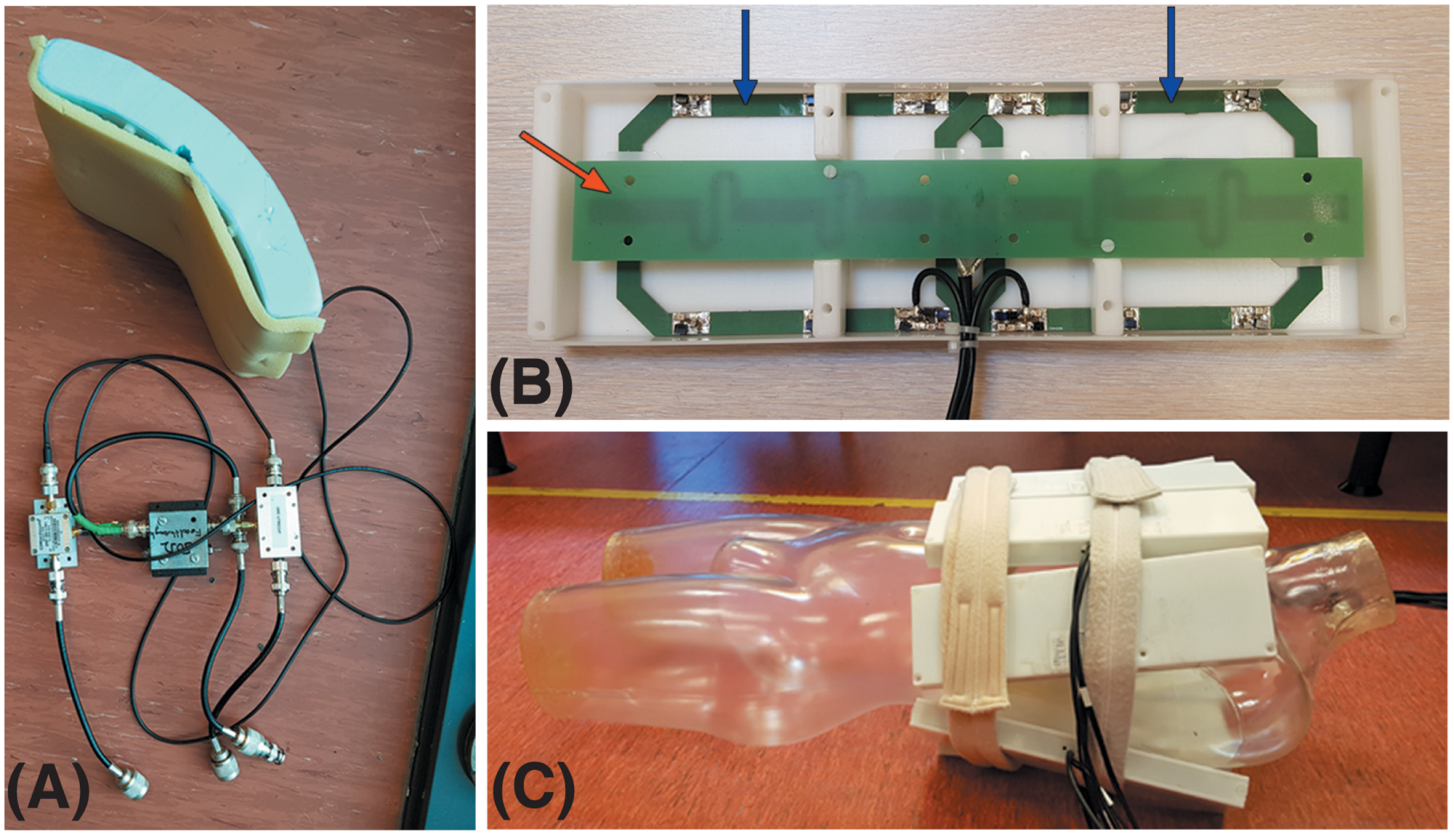
**A.)** image of the ^31^P dual‐ coil receiver from A1 with an apparent curvature to allow close contact with the body. **B.)** a view of one of the eight elements of the ^31^P 16‐channel receiver array from A2. Two ^31^P receiver coils, overlapped to improve decoupling, are denoted by blue arrows and the ^1^H meander dipole antenna for MR imaging is shown by the red arrow. **C.)** all eight elements of the ^31^P 16‐channel receiver array positioned around a plastic human mannequin representable for the *in vivo* setup for MR spectroscopic imaging of the upper torso targeting the lungs

^31^P MRSI was performed using an in‐house designed ^31^P whole body birdcage coil integrated in a 7 T MR system (Philips Healthcare, Best, Netherlands) ^22^, ^23^. The body coil, tuned at 120.6 MHz, was powered by a 25 kW amplifier (PID: 53‐S26B‐128, MKS Technologies, Shenzhen, Republic of China) resulting in a B_1_^+^ field‐magnitude of 15μT at the isocentre. Two 1H‐TxRx/31P‐Rx arrays were constructed for the experiments. Array 1 (A1) contained a ^31^P dual‐coil receiver (10 x 16 cm^2^, Figure 1A) and two fractionated ^1^H dipole antennas (30 cm) used as transceivers, both driven in quadrature mode. Array 2 (A2) contained a 16‐channel ^31^P body array with eight integrated ^1^H dipole antennas, shown in Figure 1B and C ^26^. Spectroscopic imaging data and anatomical proton images for localization were acquired in four patients using one of the two different setups.

### 2.3 Patients & setup

Four stage III‐IV non‐small cell lung carcinoma patients (ages: 53–63 years; BMI: 17.7–29.5 Kg/m^2^) were included in this feasibility study and signed informed consent prior to scanning. Two patients participated after their palliative chemo‐ and/or radiotherapy sessions and two patients participated after the first immunotherapy cycle (see Table 1 for details). Patients were scanned in supine position. Scans of two patients were acquired with the ^31^P dual coil Rx (A1) placed on the location closest to the tumor based on previously acquired clinical CT images for tumor localization. The other two patients were scanned with the ^31^P Rx array (A2), that was wrapped around the upper part of their torso. The two separate dipole antennas in A1 are positioned on the side and the top of the lung of interest. Maximum tumor dimension ranged from 25 mm to 75 mm and other clinical details per patient are shown in Table 1.

**TABLE 1:** Clinical details of each patient including relevant remarks. The body‐mass‐index (BMI) is calculated as the weight (kg) divided by the length squared (m^2^). Tumor sizes (cm^3^) represent the maximum tumor length in each direction (RL x AP x FH) and corresponding maximum volumes (cc) determined from MR and/or CT images. Treatment of non‐small cell lung carcinoma for these patients included systemic body radiation therapy (SBRT), chemoradiation and immunotherapy (Erlotinib, Pembrolizumab abbreviations: Superior vena cava (SVC)

Patient	Age (years)	BMI (kg/m^2^)	Tumor size (cm^3^|cc)		Therapy	Remarks
#1	59	20.4	7.25 x 1.75 x 1.00|	12.69	Seq. Chemoradiation	Stent in SVC close to tumor
#2	60	17.7	3.75 x 4.80 x 2.00|	36.00	Thoracic SBRT	‐
#3	63	24.2	3.60 x 3.20 x 3.60|	41.47	Erlotinib	‐
#4	53	29.5	3.60 x 3.00 x 2.50|	27.00	Pembrolizumab	‐

### 2.4 MR data acquisition

No B_0_ shimming was performed nor was the ^31^P B_1_^+^ calibration. Phosphorus (^31^P) spectra were acquired using a 3D ^31^P acquisition weighted CSI protocol including elliptical k‐space sampling. Excitation was performed using rectangular RF pulses only and the carrier frequency was set to PCr. The isotropic resolution ranged from 20 to 30 mm and other parameters are summarized in Table 2 ^24^.

**TABLE 2:** 3D ^31^P CSI protocol parameters for each patient including the resolution, matrix size (RL x AP x FH), repetition time (TR), echo time (TE), flip angle, bandwidth (BW), number of sampled averages (NSA), number of sample points, scan duration and number of ^31^P receiver channels (#Rx).). **α)** nominal voxel volume corrected for weighted acquisition^24^

**Patient**	Resolution nominal (mm^3^)|real (cc)^α^	Matrix (RL x AP x FH)	TR/TE (ms)	Flip angle	BW (Hz)	NSA	Sample points	Scan duration (min: Sec)	**#Rx (** ^**31**^ **P)**
#1	26 x 26 x 26| 31	7 x 5 x 6	60/0.54	20°	4800	320	256	23:00	2
#2	20 x 20 x 20| 14	12 x 7 x 9	60/0.54	12°	4800	80	256	23:00	2
#3	30 x 30 x 30| 48	12 x 6 x 6	60/0.51	9°	5000	60	256	25:55	16
#4	30 x 30 x 30| 48	15 x 11 x 8	60/0.44	10°	4800	60	256	28:15	16

### 2.5 Data processing

Spectroscopic data from the 3D CSI protocol were processed in Matlab 2018b (The Mathworks Inc., Natick, MA) using an open source in‐house designed processing tool (CSIgui v1.1, http://www.csigui.tk, April 2019). ^31^P spectroscopy data were averaged and spatially filtered using a 3D hamming window followed by an inverse Fourier transformation to the spatial domain. All free induction decays (FID) were apodized using a 24 Hz gaussian filter and zero filled to 512 samples. Coil data was combined using the whitened singular value decomposition (WSVD) algorithm as reported by Rodgers et al ^27^. Zeroth order phase correction was applied automatically, and first order phase correction was applied manually, thereafter. No additional nor aesthetic baseline corrections were performed. Spectra from tumors exceeding the voxel resolution were aligned to the metabolite peak with the highest SNR followed by averaging, excluding voxels with a 50% or less partial tumor tissue volume on available MR images. The SNR of metabolites was calculated using equation [2] with S_max_, the real part of the maximum signal intensity and the noise defined as the absolute standard deviation of the last 50 samples points of the spectrum.

(2) $$\mathrm{SNR}=\frac{\text{real}\left(\;S_{\mathrm{Max}}\right)}{|\mathrm{std}\left(S_{\text{noise}}\right)|}$$

## 3 RESULTS

**FIGURE 2:**
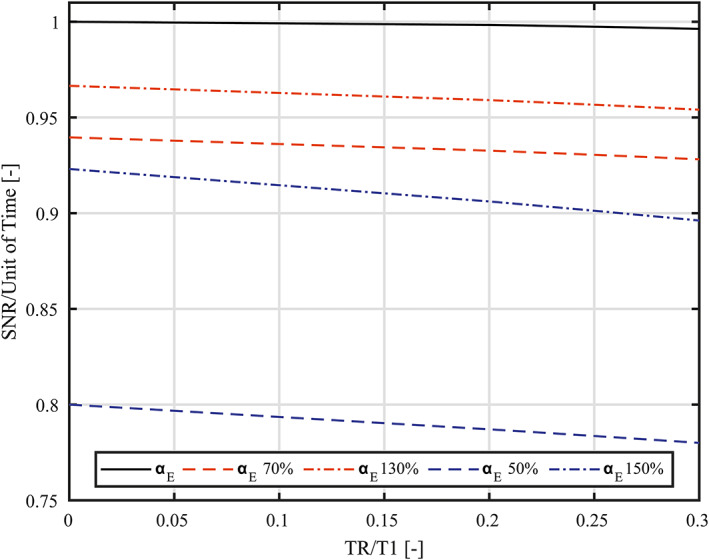
Simulation of the SNR per unit of time for the 3D ^31^P spectroscopic imaging at Ernst angle (α_E_) and with a 30% and 50% deviation for the TR/T_1_ ratio ranging from 10^−6^ to 0.3. The SNR per unit of time at α_E_ is marked by the solid black line, the increased and decreased angles for both the 30% (red) and 50% (blue) deviations are displayed as dashed and dash‐dotted lines respectively

Simulations resulted in a maximum decrease of 8% in SNR per unit of time within the used TR/T_1_ range for + and − 30% deviating flip angles, as can be seen in Figure 2. In addition, the α_E_ + 30% variation showed a lower decrease in SNR per unit of time compared to the α_E_ ‐ 30% variation. A similar trend is seen for a α_E_ ± 50% variation showing a maximum decrease of 23% in SNR per unit of time within the same TR/T1 range for the α ‐ 50% variation. According to the B_1_ maps available for the ^31^P body coil, we could expect a maximum of 30% deviation in flip angle in the *in‐vivo* measurements using equal power settings between subjects and, in addition, a maximum decrease in SNR per unit of time of less than 6% is seen for the TR/T_1_‐ratios range that corresponds to the ^31^P metabolites of interest (0.009; 0.13) and the proposed protocol TR (60 ms) ^23^.

**FIGURE 3:**
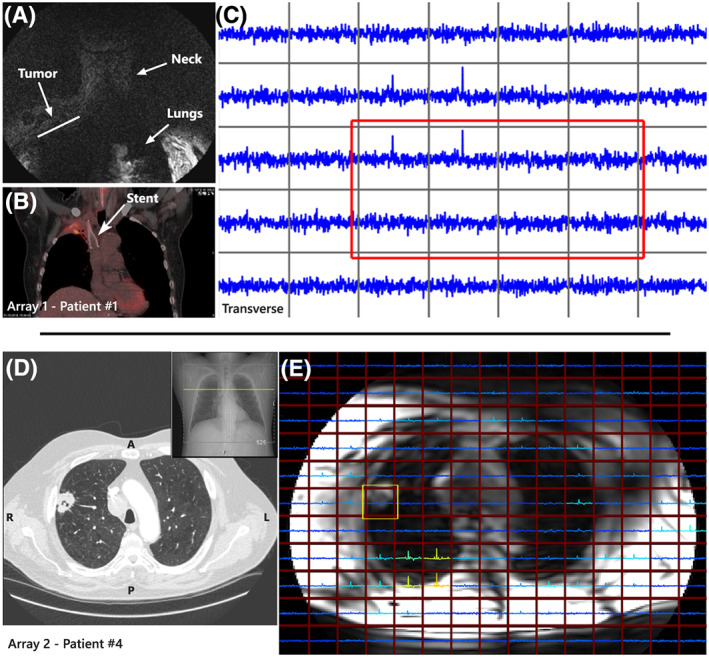
**A)** Coronal MR image including labels for the tumor, neck and lungs plus **B)** a coronal CT image with PET scan overlay, both from patient #1 and used for tumor localization. **C)** Single transverse slice of the 3D spectroscopic imaging data from patient #1 with the tumor voxels indicated by the red rectangle. **D)** Transverse and coronal CT images from patient #4 for tumor localization and planning. **E)** the MR image from patient #4 with an overlay of a single slice of the 3D spectroscopic imaging data. Tumor voxel is highlighted by the yellow rectangle

All patients were imaged within an hour of scan time with one of the two setups. Positioning the ^1^H transmit coils for patient #1 was limited due to a stent in the superior vena cava (SVC) located close to the tumor. No other patient related difficulties were experienced during the scan sessions. Images obtained with the dipole antenna in A1 were adequate for tumor localization and planning (Figure 3A), when tumor location was known from previous CT images (Figure 3B).

Images and tumor localization using A2 were improved compared to A1 as is depicted in Figure 3A and 3E. Spectroscopic imaging acquisitions could be obtained with both setups as shown in figures 3C & E, where a single spectroscopy slice from the 3D imaging set for patient #1 and #4 are shown respectively. Tumor voxels in the slice used for averaging are marked by the red and yellow rectangles, showing 8 out of 20 voxels for patient #1 and all tumor voxels for patient #4. In addition, the signal intensity of the voxels located at the posterior side of the patient in the spectroscopic imaging array in Figure 3E have higher SNR compared to the anterior side.

**FIGURE 4:**
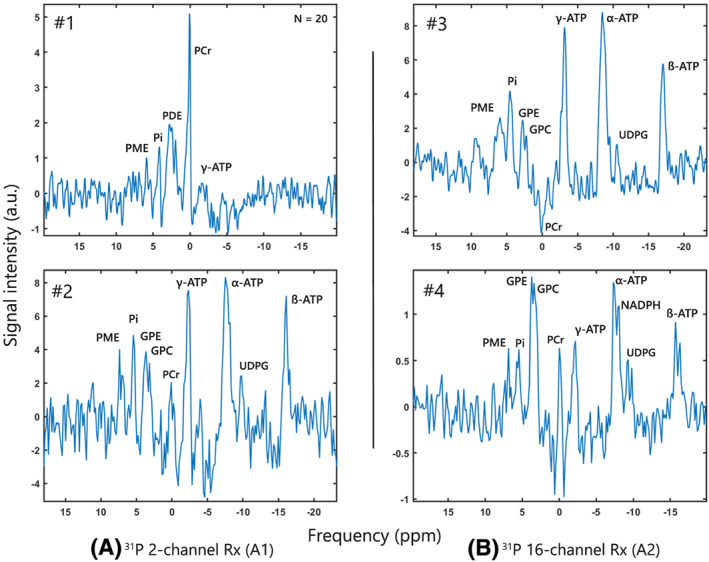
Spectra of lung tumor tissue for all four patients acquired with the ^31^P chemical shift imaging protocol using **a)** A1, the ^31^P dual coil Rx and **B)** A2, the ^31^P 16‐channel Rx array. Phosphomonoesters (PME), phosphodiesters (PDE), glycerophosphoethanolamine (GPE) plus glycerophosphocholine (GPC), inorganic phosphate (pi), phosphocreatine (PCr) and the α‐, β‐ and γ‐ ATP resonances are labelled where applicable. The number of tumor voxels used for averaging is denoted by N in the right top corner of the spectrum except for single voxel spectra. Notice the increase in PDE with respect to PME in patient #4 that might indicate tumor response to immunotherapy

Obtained ^31^P lung carcinoma spectra were acquired with high SNR for PCr (9.5) and the ATP resonances (>4.7) using A1, the ^31^P dual‐coil Rx (Figure 4A) and with high SNR ranging from 3.9 (PME) to 13.2 (α‐ATP) using A2, the ^31^P 16‐channel Rx array (Figure 4B). It allowed discrimination of PME, Pi, PDE, PCr in all patients, the three ATP resonances and UDPG in all subjects except for patient #1 and NADPH in patient #4. Moreover, the SNR of the phospholipid‐ and energy‐ metabolites was found higher with A2 compared to A1. The lack of B_0_ shimming and partial volume effects over the large field of view is visible in the spectra with measured linewidths ranging from 0.20 ppm to 1.1 ppm after apodization when using either coil setup.

## 4 DISCUSSION

3D ^31^P spectroscopic imaging was successfully obtained in four lung carcinoma patients with either the ^31^P dual‐coil receiver or the 16‐channel receiver array in combination with the integrated ^31^P body coil at 7 tesla. Both Rx setups allowed the acquisition of phosphorous metabolic information from the lung carcinoma via a non‐invasive method, while targeting the full organ for evaluation. The increased spectral resolution at the ultra‐high magnetic field strength of 7 T allowed differentiation of multiple phosphorous metabolites related to cell membrane and energy metabolism. A minimal decrease in SNR per unit of time was apparent from the simulations performed to study the effect of a +/−30% deviation from the Ernst angle due to the lack of individual body coil power calibrations in this patient population. This minimal SNR loss of at maximum 8% allowed for 3D fast spectroscopic imaging with short TR and low flip angle excitation without time‐consuming calibration steps during the scan session.

Increasing the number of receiver coils improved the field of view coverage of ^31^P MRS images expanding the available metabolic information over a larger field of view. This agrees with previous demonstrations in literature ^28^. In addition, the SNR increase gained with the 16‐channel receiver array used in patient #3 and #4 not only allowed discrimination of PME, Pi, GPE, GPC, PCr, ATP (with α‐, ß‐ & γ‐resonances) and uridine‐diphosphate glucose (UDPG) as with the dual‐coil receiver but also nicotinamide‐adenine dinucleotide phosphate (NADPH) in patient #4. UPDG is a known liver metabolite and indicates minor liver signal contamination, however SNR was insignificant (SNR < 3) ^5^. NADPH (SNR > 3) however, though also found in the liver, is a cofactor involved with anabolic reactions, already linked to tumor tissue ^29^. In addition, the highest SNR of the dual‐coil receiver was measured for PCr (Figure 4A, Patient #1) which is not directly associated with tumor tissue, but rather muscle tissue ^5^. This can be explained by signal contamination from chest muscle signals contained in neighbouring voxels that bled in the tumor voxel location due to the small field of view of this patient, which excluded the full body circumference, in combination with the relatively large voxel size. Additional averaging of the 20 voxels also increased signal contamination in this patient but was required to regain the SNR of the spectrum. Spectra acquired with the 16ch Rx still show signal contamination, as can be seen by the remaining PCr peaks, though to a lesser extent than the first patient and even in opposite phase (Figure 4B, patient #3). The lung and tumor morphology itself may already minimize signal contamination from neighboring voxels as tissue density in healthy lung tissue is, compared to other areas in the body, extremely low. Further protocol development could minimize signal contamination within a short time frame by increasing spatial voxel resolution or reducing point spread by more complex k‐space weightings and filtering ^24^. Another strategy could be the use of selective pulses to fully eliminate specific tissue signal such as the one coming from the muscles.

The top part of the torso, especially at the collar bones, limits proper positioning of the top elements of the receiver array. The eight rigid elements of the array lack the body‐shaped curvature, disfavouring coil loading and resulting in a suboptimal receive fields for these coils. This can be seen by the increased SNR at the posterior side of the patient compared to the anterior voxels shown in Figure 3C. Additional suboptimal coil combinations using the WSVD algorithm could also disfavour the SNR gradient over the spectroscopic image.

Moreover, in the presence of large susceptibility differences, such as the lung itself, implants and the moving heart, spectral quality is surprisingly adequate for the distinction of the metabolites, even without B_0_ shimming. Resulting B_0_ homogeneity was adequate with a spectral linewidth ranging between 0.2 and 1.1 ppm. This B_0_ non‐uniformity is much less critical than for ^1^H MRS as the spectral separation between metabolites (i.e. PME versus PDE) is substantial (i.e. >3.5 ppm) at 7 T. However, it should still be noted that the B0 field uniformity can be highly variable both spatially as temporally. In our previous study we simulated spatiotemporal magnetic field uniformity, which at worst case conditions (i.e. at diaphragm comparing fully inhaled versus exhaled condition) could be up to 3 ppm ^30^. Either avoiding inclusion of subjects with tumors in locations of such severe non uniformities or using new means of local shim coils that can mitigate these distortions may be required ^31^. In addition, shimming procedures could be performed within a breath‐hold and when combined with gating it is expected to improve linewidth, possibly increasing sensitivity to allow detections of the individual PME and PDE ^32^.

In this study we have altered the flip angle between subjects. As prior knowledge about signal levels was unknown, we started by focusing on ATP, therefore setting the flip angle to 20°.After confirming observation of phospholipids, the angle was set to 9° (i.e. Ernst angle for PME and PDE). Finally, we completed the protocol by small over‐tipping to also consider SNR of other metabolites that all have a shorter T_1_. Note that the spectra are obtained with T_1_ weighting, so altered peak ratios can be caused by concentration differences, but also by alterations in T_1_. To extract the T_1_ dependence, subject specific T_1_ knowledge could be obtained by acquiring the same scan twice albeit with a different flip angle.

## 5 CONCLUSION

We conclude that ^31^P MRSI in lung carcinoma is feasible at 7 T. Employing large receiver arrays that can cover the whole torso, improves the field of view coverage allowing full organ ^31^P‐MRSI acquisition. With only minor signal contamination to overcome, ^31^P MRSI shows great potential as tumor biomarker for treatment response monitoring in lung cancer.
